# Dielectrophoresis-Enhanced Microfluidic Device with Membrane Filter for Efficient Microparticle Concentration and Optical Detection

**DOI:** 10.3390/mi16020158

**Published:** 2025-01-29

**Authors:** Young-Ho Nam, Seung-Ki Lee, Jae-Hyoung Park

**Affiliations:** 1 Department of Electronics and Electrical Engineering, Dankook University, Yongin 16890, Republic of Korea; nyh7241@gmail.com; 2 Department of Semiconductor Convergence Engineering, Dankook University, Yongin 16890, Republic of Korea; skilee@dankook.ac.kr

**Keywords:** microfluidics, dielectrophoresis, membrane filter, microparticle concentration

## Abstract

This paper presents a novel microfluidic device that integrates dielectrophoresis (DEP) forces with a membrane filter to concentrate and trap microparticles in a narrow region for enhanced optical analysis. The device combines the broad particle capture capability of a membrane filter with the precision of DEP to focus particles in regions optimized for optical measurements. The device features transparent indium tin oxide (ITO) top electrodes on a glass substrate and gold (Au) bottom electrodes patterned on a small area of the membrane filter, with spacers to control the gaps between the electrodes. This configuration enables precise particle concentration at a specific location and facilitates real-time optical detection. Experiments using 0.8 μm fluorescent polystyrene (PS) beads and *Escherichia coli* (*E. coli*) bacteria demonstrated effective particle trapping and concentration, with fluorescence intensity increasing proportionally to particle concentration. The application of DEP forces in a small region of the membrane filter resulted in a significant enhancement of fluorescence intensity, showcasing the effectiveness of the DEP-enhanced design for improving particle concentration and optical measurement sensitivity. The device also showed promising potential for bacterial detection, particularly with *E. coli*, by achieving a linear increase in fluorescence intensity with increasing bacterial concentration. These results highlight the device’s potential for precise and efficient microparticle concentration and detection.

## 1. Introduction

The concentration and trapping of microparticles are critical challenges in various fields, including environmental monitoring (e.g., microplastics) and disease diagnostics, where the detection of low-concentration biomarkers, pathogens, or microparticles in samples is essential [1,2,3,4,5,6,7]. Microfluidic chips have emerged as effective platforms for these applications due to their capability for precise particle manipulation and high-sensitivity detection. Concentration and trapping technologies utilizing microfluidic chips enable the efficient separation and analysis of target particles from small-volume samples, offering advantages such as speed and cost-effectiveness compared to conventional concentration methods.

Microparticle trapping methods using microfluidic chips can be broadly categorized into active and passive approaches. Active methods involve the use of external energy sources to manipulate microparticles. One such active method is acoustic microfluidics, with surface acoustic wave (SAW)-based techniques being a notable example. In this approach, interdigital transducers (IDTs) are fabricated on a piezoelectric substrate to generate surface acoustic waves. These waves interact with microparticles to concentrate them in a specific region [8,9,10,11,12,13,14,15,16]. On the other hand, it forms acoustic streaming around the structure within the channel and uses this to trap particles around the structure [17,18]. These methods are limited by low spatial resolution, which restricts their precision. Optical tweezers utilize a powerful laser beam to manipulate microparticles or cells with nanometer-level precision [19,20,21,22,23,24,25]. This method is well suited for handling individual cells. However, the intense laser can potentially damage cells and is less effective for simultaneously manipulating multiple particles. Dielectrophoresis (DEP) refers to the motion of polarized particles induced by spatially non-uniform electric fields [26,27,28,29,30,31]. This method is applicable to cells and microparticles, enabling the trapping of multiple particles with relatively high precision. Active techniques such as DEP, optical tweezers, and acoustic microfluidics allow for effective particle concentration and trapping in a non-contact and label-free manner.

In contrast, passive-type devices operate solely with a pump, without the need for external energy sources, and typically feature relatively simple structures. Hydrodynamic trapping is a representative passive microparticle trapping technique [32,33,34,35,36,37,38,39]. This method controls fluid flow through the geometric design of microfluidic channels, ensuring that particles are trapped at specific locations. To achieve this, structures such as small obstacles or traps (e.g., grooves or holes) are incorporated into the channel to modulate fluid resistance. The fluid naturally flows along the path of least resistance, guiding particles to regions with reduced flow velocity, where they become trapped. Another widely used passive method involves the use of membrane filters, one of the most straightforward and intuitive approaches for capturing microparticles [40,41,42,43,44,45]. By adjusting the pore size of the membrane, this technique allows for the selective trapping of desired particles. While these passive methods are simple and effective for particle trapping, they are prone to clogging when a significant number of particles accumulate in filter pores or structural elements. When the membrane filter becomes clogged with particles, the hydrodynamic resistance increases, leading to changes in flow velocity, flow rate, and pressure. These changes can degrade the performance of the microfluidic system and cause unexpected behavior. To prevent such issues, the area of the membrane filter is often increased. However, increasing the filter area causes microparticles to spread over a larger region, making the measurement of optical signals more challenging. Additionally, clogging can reduce the device’s lifespan and frequently occurs when processing high-concentration samples, representing a key limitation of such approaches.

In this study, we present a novel microfluidic device that integrates a DEP-based particle trapping mechanism within a membrane filter, aimed at concentrating particles in a narrow region for optical measurement. The device combines a mechanical membrane filter, capable of capturing particles over a wide area, with electrodes designed to generate DEP forces that concentrate particles in a region optimized for optical analysis. This approach seeks to achieve high particle recovery efficiency across the membrane filter and enhanced particle concentration in the narrow region through DEP forces. Furthermore, the design addresses the clogging issues commonly encountered with traditional membrane filters. The device incorporates ITO electrodes patterned on a glass substrate and Au electrodes patterned on the membrane filter in a 3D configuration, which facilitates the generation of DEP forces. PDMS spacers are employed to control the gap between the electrodes and to function as channel walls within the microfluidic system. By utilizing DEP, microparticles are trapped within designated areas of the membrane filter, enabling the real-time optical monitoring of the trapped particles through the transparent glass substrate and ITO electrodes. Particle concentration experiments using PS beads were conducted under varying conditions, including voltage and particle concentration, to assess the device’s performance. These experiments were further extended to bacteria, demonstrating the device’s potential for bacterial detection.

## 2. Design

**Figure 1:**
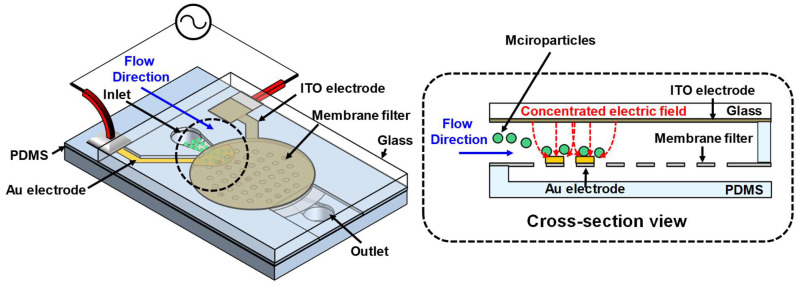
Schematic diagram of the microfluidic device integrating a DEP-based particle concentration mechanism within a membrane filter.

Figure 1 illustrates a schematic and cross-sectional view of the microfluidic device. The microfluidic device is designed to utilize positive dielectrophoresis (pDEP) to concentrate microparticles in specific regions. The lower electrode is formed only in certain areas of the membrane filter, while the upper electrode is designed to be larger than the lower electrode to focus the electric field on the lower electrode. When a solution containing microparticles is injected through the inlet, it flows through the channel between the upper and lower electrodes, where an AC signal is applied. In this process, the non-uniform electric field induces polarization of non-polar particles through the polarization effect, and the polarized particles are moved by the DEP force.

The time-averaged DEP force acting on a spherical particle can be expressed as follows:

(1) $$F_{DEP}=2\pi \cdot \varepsilon_{m}\cdot Re\left(f_{CM}\right)\cdot r^{3}\cdot \nabla {\left|E\right|}^{2}$$

where $\varepsilon_{m}$ is the absolute permittivity of the medium, r is the radius of the particle, and $\nabla {ΙEΙ}^{2}$ is the gradient of the square of the applied electric field. RefCM is the real component of the complex Clausius (CM) factor, given by

(2) fCM=εp*−εm*εp*+2εm*, where ε*=ε−jσω 

where $\varepsilon^{*}$ represents the complex permittivity and p and m refer to the particle and medium, respectively. σ is complex conductivity, ω is the angular frequency (ω=2πf) of the applied electric field, and $j=\sqrt{-1}$. The value of RefCM lies between −0.5 and 1. If RefCM > 0, the particle experiences a pDEP force. If RefCM < 0, the particle experiences a negative DEP force (nDEP). We adjusted the frequency to apply a pDEP force to the microparticles and designed the device to trap microparticles at the lower electrode, where the electric field is concentrated. Additionally, a transparent ITO electrode was used as the upper electrode on the glass substrate to enable real-time fluorescence observation.

**Figure 2:**
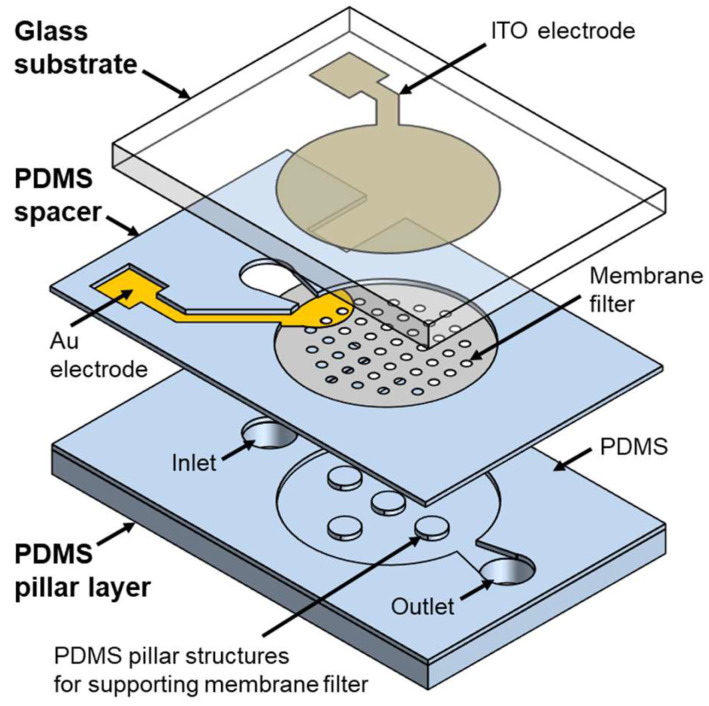
Exploded view of microfluidic device.

Figure 2 illustrates an exploded view of the DEP device integrated with a membrane filter. The device is composed of several key components: a glass substrate, PDMS spacer, membrane filter, and a PDMS pillar layer. The glass substrate has a thickness of 500 μm, with ITO used as the top electrode. The ITO electrode is designed as a circular shape with a 6 mm diameter, slightly larger than the membrane filter. The polycarbonate track etched (PCTE) membrane filter, with a 5 mm diameter and 400 nm pores (111107, Whatman, Maidstone, Kent, UK), is capable of capturing bacteria such as *Escherichia coli* (*E. coli*). To maximize particle concentration through DEP forces, the lower electrode on the membrane filter is positioned close to the point where the particles are released towards the filter. Furthermore, the lower electrode is designed with a semicircular shape to ensure that the particles experience the same distance of influence from the DEP forces as the particles traverse the electrode area on the filter. The lower electrode is fabricated by patterning gold (Au) with a 500 μm diameter. The PDMS spacer, with a thickness of 40 μm, is bonded to the membrane filter and adjusts the gap between the gold electrode on the filter and the ITO electrode on the glass substrate. In addition, the spacer serves as the channel wall for the microfluidic device, guiding the fluid flow. The PDMS pillar layer incorporates supporting column structures that stabilize the filter and create channels for the discharge of solutions.

## 3. Materials and Methods

### 3.1. Materials

The microparticles used in the trapping experiments were polystyrene (PS) beads and bacteria. The PS beads were 0.8 μm fluorescent particles labeled with FITC (F1CP-08-2, Spherotech, Lake Forest, IL, USA). The bacteria used were KACC 11598 *E. coli* O157:H7, purchased from the Korean Agricultural Culture Collection (KACC). The *E. coli* were cultured in 20 mL of sterile tryptic soy broth (TSB) solution and incubated in a shaking incubator at 37 °C for 12 h. After incubation, the solution was centrifuged at 1977× *g* and 4 °C to isolate the *E. coli*, which was then resuspended in phosphate-buffered saline (PBS) with a pH of 7.4. The *E. coli* were stained with SYTO9 Green Fluorescent Nucleic Acid Stains, purchased from Thermo Fisher Scientific. The PS bead and *E. coli* solutions were serially diluted in PBS to concentrations of 10⁶ particles/mL, 10^5^ particles/mL, 10^4^ particles/mL, 10^3^ particles/mL, and 10^2^ particles/mL.

### 3.2. Fabrication

**Figure 3:**
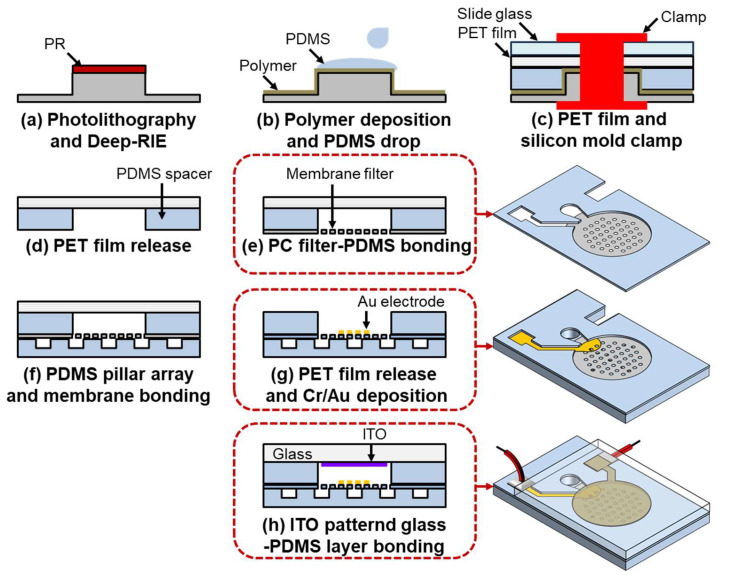
Fabrication diagram of the microfluidic device: (**a**) Photolithography process and Deep-RIE for fabricating the silicon mold. (**b**) Polymer deposition to reduce adhesion and PDMS drop-casting. (**c**) Clamping the PET film and silicon mold for spacer fabrication. (**d**) Peeling off the PET film. (**e**) Bonding the PC filter with PDMS. (**f**) Bonding the PDMS pillar array with the membrane filter. (**g**) Peeling off the PET film and electrode deposition using a shadow mask. (**h**) Oxygen plasma bonding between the ITO electrode-formed glass substrate and the PDMS layer.

Figure 3 depicts the fabrication process of the DEP device integrated with a membrane filter. The process begins with the fabrication of the silicon mold, which is used to create the PDMS spacer. A p-type <100> silicon wafer undergoes an SPM cleaning process, followed by the deposition of a 2 μm thick layer of positive photoresist. The photoresist is patterned using photolithography, leaving the resist only in the fluid channel areas. A deep reactive ion etching (DRIE) process is then performed, using the patterned photoresist as a mask, to etch the silicon substrate to a depth of 40 μm, which determines the channel depth and the gap between the electrodes. To reduce adhesion between the PDMS and silicon, a polymer layer is deposited using C_4_F_8_ gas, and the silicon wafer is diced into 4 cm × 2 cm pieces. Subsequently, 1 mL of PDMS mixed with a curing agent at a 10:1 ratio is poured onto the silicon mold. A 2 cm × 2 cm polyethylene terephthalate (PET) film is placed on top of the PDMS, followed by a slide glass. The assembly is clamped with a jig and baked at 85 °C for 2 h to cure the PDMS. After curing, the silicon mold and slide glass are removed. The adhesion between the PDMS and the PET film is stronger than that between the PDMS and the silicon mold, resulting in the PDMS spacer being bonded to the PET film, as shown in Figure 3d.

The subsequent step involves bonding the PDMS spacer to the membrane filter using a stamping method with diluted PDMS. Initially, PDMS is mixed with toluene at a 1:1 ratio and spin-coated onto a slide glass at 4000 RPM. The PET film with the attached spacer is then stamped onto the slide glass, allowing the diluted PDMS to adhere exclusively to the unbonded side of the PDMS spacer. Next, a 5 mm × 5 mm PC membrane filter is affixed to the PDMS-coated spacer, and the assembly is cured in an oven at 85 °C for 2 h to complete the bonding process. The filter-attached spacer is subsequently bonded to the PDMS pillar layer after oxygen plasma surface treatment. Both bonding surfaces are treated with a plasma generator for 30 s. The treated surfaces are then aligned and baked in an oven at 85 °C for 1 h, after which the PET film is removed. Due to the weaker adhesion between the PET film and PDMS spacer compared to the bond between the PDMS spacer and the PDMS pillar layer, the PDMS spacer with the attached filter remains bonded to the PDMS pillar layer, while the PET film is detached.

The next step in the fabrication process is electrode patterning. Both the bottom Au and top ITO electrodes are patterned using shadow masks. For the bottom electrode on the membrane filter, precise alignment between the PDMS spacer and the shadow mask is crucial, which is achieved using an x, y, z stage and a microscope. Subsequently, 100/1000 Å of Cr/Au is deposited onto the membrane filter using the aligned shadow mask. The top ITO electrode is patterned on a glass substrate with a deposition thickness of 200 nm. After patterning, the substrate is diced into 2 cm × 2 cm pieces. The final step involves bonding the glass substrate to the PDMS structure. The glass substrate and PDMS spacer are bonded after plasma treatment, where both bonding surfaces are treated with a plasma generator for 30 s, then joined and baked at 85 °C for 1 h to complete the device fabrication.

### 3.3. Experimental Setup

**Figure 4:**
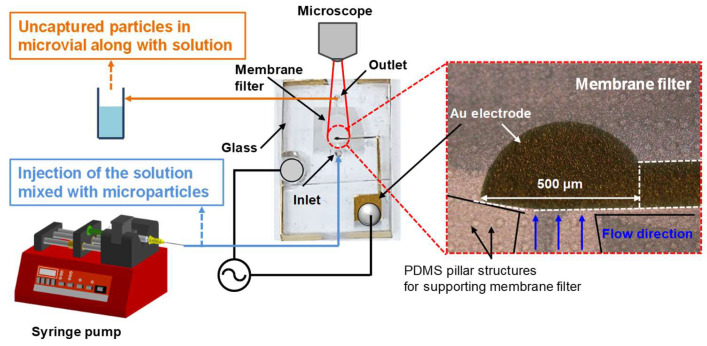
Photographs of the fabricated microfluidic device and experimental setup for microparticle concentration within the microfluidic device.

Figure 4 illustrates the experimental setup for concentrating microparticles within the microfluidic device, and a photograph of the fabricated microfluidic device. The size of the fabricated microfluidic device is 4 cm × 2 cm with a thickness of 0.5 cm. The microparticles utilized in the experiments were 0.8 μm PS beads and *E. coli*, both of which were diluted in PBS. The microparticle trapping experiments were conducted on a microscope stage, with fluorescence imaging performed using an Olympus BX53 fluorescence microscope. Fluorescence images were captured under consistent light intensity and exposure time conditions. The fluorescence intensity of the captured images was quantified using ImageJ software. All solutions used in the experiments were accurately controlled and injected using a syringe pump (NE-300, New Era Pump Systems, East Farmingdale, NY, USA). The AC signals applied to the two electrodes of the microfluidic chip were generated by a function generator (DG1022, RIGOL, Suzhou, China) and monitored in real-time using an oscilloscope (DS1102E, RIGOL Suzhou, China).

## 4. Results and Discussion

**Figure 5:**
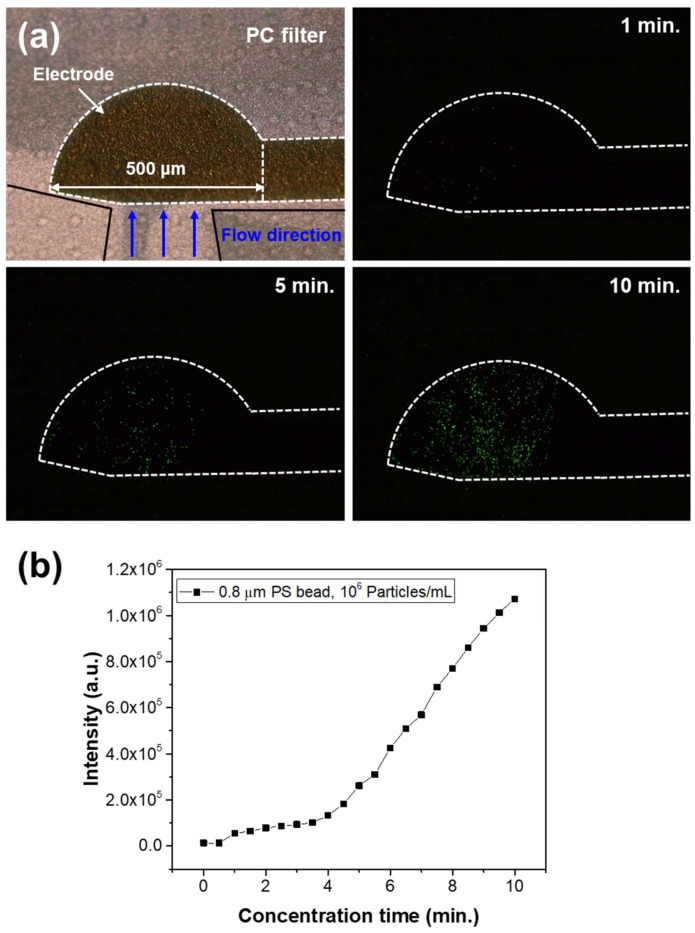
(**a**) Fluorescence images at different particle concentration times. (**b**) Fluorescence intensity measurements as a function of concentration time.

Experiments were conducted to evaluate the microparticle trapping performance of the microfluidic chip. An AC signal of 20 V_pp_ at 10 kHz was applied to the two electrodes of the chip. Under these conditions, a solution containing 0.8 μm fluorescent beads at a concentration of 10⁶ particles/mL was injected at a flow rate of 1.0 μL/min for 10 min, and the changes in fluorescence signal were measured over time. Figure 5a presents fluorescence images of the membrane filter at different particle concentration times. The white lines in the images indicate the boundaries of the electrode patterns on the membrane filter, with the interior representing the electrode area. The fluorescence images show that as the trapping time increased, a greater number of fluorescent particles accumulated along the electrode boundaries and within the electrode area. The fluorescence intensity of the images was analyzed using ImageJ software. To ensure data consistency, the intensity of the fluorescence light source and the camera exposure time were kept constant during imaging. Fluorescence intensity was measured at 30 s intervals, and the results are shown in Figure 5b. The graph illustrates a linear increase in fluorescence signal over time, with an average fluorescence signal increase of 1.1 × 10^5^ per minute.

**Figure 6:**
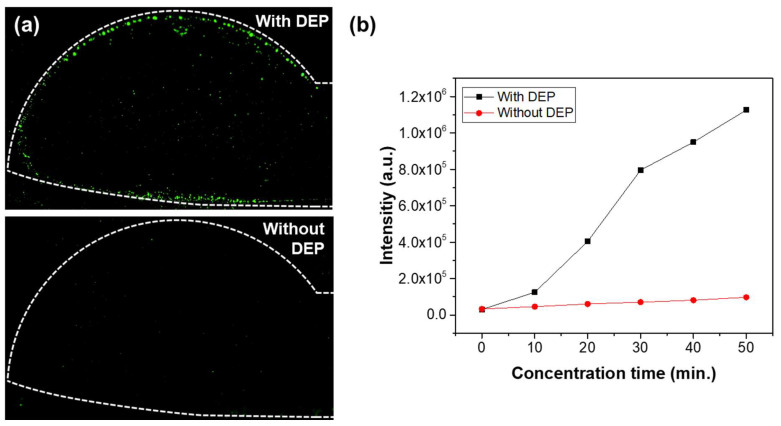
(**a**) Fluorescence images with and without the application of DEP force. (**b**) Comparison of fluorescence intensity with and without the application of AC voltage.

To evaluate the impact of DEP-based particle concentration in a small electrode region, we conducted comparative experiments with and without the application of an AC signal. A solution containing 0.8 μm fluorescent beads at a concentration of 10⁵ particles/mL was injected at a flow rate of 1.0 μL/min for 50 min. An AC signal of 20 V_pp_ at 10 kHz was applied in the experimental group, while the control group was tested without the AC signal. Figure 6a shows fluorescence images of the membrane filter under both conditions, highlighting a distinct difference that is clearly observable using the fluorescence microscope. The white dashed lines in the figure represent the edges of the lower electrode, and as shown in the image, the particles are concentrated in the electrode region, where green fluorescence is observed. It can be observed that the fluorescence intensity is significantly higher around the electrode borders. This is due to the strong electric field concentration effect at the edges of the electrode, which generates a relatively large DEP force, leading to more particles being trapped in that area. Figure 6b compares the fluorescence signals with and without the AC signal. The particles were trapped for a total of 50 min, and the green fluorescence intensity within the image region was measured at 10 min intervals after applying the voltage, using ImageJ software. While the fluorescence intensity increased over time in both conditions, the experiment with the AC signal exhibited fluorescence intensities over 10 times greater than those observed without the AC signal. These findings demonstrate that integrating electrodes generating DEP forces onto the membrane filter effectively concentrates particles at high density in the narrow region where optical measurements are conducted, thereby significantly enhancing the optical measurement sensitivity compared to particle concentration using only a membrane filter over a large area.

The recovery rate of particle capture can be quantified using the following equation, which is based on the ratio of the concentration of particles that escaped without being captured to the total concentration of injected particles:

(3) $$\mathrm{Recovery}\;\mathrm{rate}\;\left(\%\right)=1-\frac{concentration_{outlet}}{concentration_{inlet}}$$

**Figure 7:**
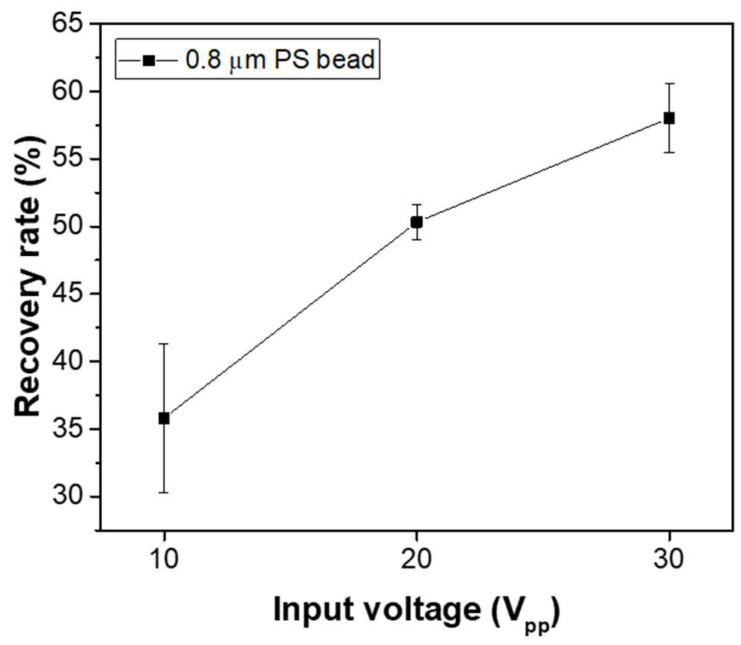
Recovery rate as a function of applied voltage under conditions with only DEP forces, excluding the membrane filter.

In the case of the membrane filter utilized in the microfluidic device developed in this study, the concentration of particles that passed through the filter pores without capture was nearly negligible throughout the experiment, yielding a recovery rate close to 100%. In comparison, the particle recovery rate using only DEP forces was also evaluated. For this, a device with the same electrode structure but without the membrane filter was fabricated, where particles injected into the inlet were captured by DEP forces within the electrode region. Particles not captured by DEP forces were allowed to exit through the channel outlet, and their concentration was measured to assess the recovery rate under conditions with only DEP forces. Figure 7 presents the capture efficiency as a function of the input voltage applied to the electrodes. The concentration process lasted for 50 min, with 0.8 μm fluorescent beads at a concentration of 10^5^ particles/mL injected at a flow rate of 1.0 μL/mL. The input signal frequency was 10 kHz, and the applied input voltages were 10 V_pp_, 20 V_pp_, and 30 V_pp_. The experimental results revealed recovery rates of 35.08 ± 5.52%, 50.32 ± 1.23%, and 58.01 ± 2.53% for each voltage, respectively. These rates were lower than those achieved with the membrane filter, indicating that DEP forces alone provided less efficient particle capture. However, as shown in the results of Figure 6, when DEP forces were applied to a small region of the membrane filter, the fluorescence intensities increased more than 10 times during the 50 min concentration period. This suggests that when particles are captured solely by DEP forces, many particles are lost due to horizontal fluid flow. In contrast, applying DEP forces to the membrane filter induces vertical flow through the filter pores, which allows the particles to be captured by the pores. The combined effect of particle capture through the pores and the confinement of particles within the region by DEP forces results in a significant increase in particle concentration in the electrode region.

**Figure 8:**
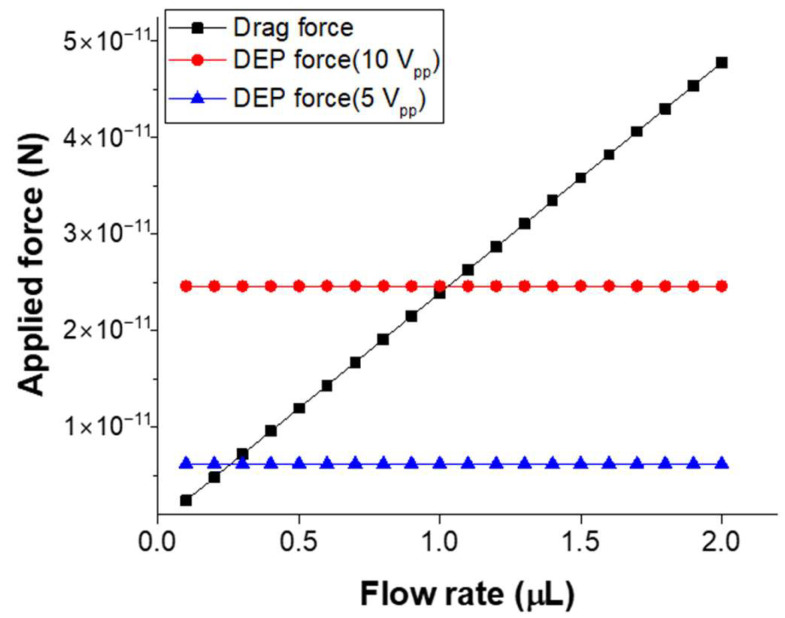
Comparison of the drag force on the particles with the applied DEP force.

In the microfluidic device developed in this study, the force that most significantly influences the particles during fluid flow is the drag force, resulting from the friction between the particles and the surrounding fluid. This drag force varies with the flow rate, and since it affects the motion of the particles near the electrodes, a comparison of the drag force and DEP force was conducted as a function of flow rate and applied voltage. This comparison was used to determine the optimal flow rate and voltage for fluorescence intensity detection based on particle concentration after trapping. The drag force, which dominates under low Reynolds number and viscous flow conditions within the microfluidic device, was calculated using Stokes’ law:

F = 6πηrv

where η is the fluid viscosity, r is the particle radius, and v is the fluid velocity. The DEP force was calculated by simulating the electric field between the upper and lower electrodes, with a 40 μm gap and the channel filled with PBS. Figure 8 presents the calculated drag force acting on 0.8 μm particles and the DEP force under varying applied voltages, with flow rates ranging from 0.1 μL/min to 2.0 μL/min. The voltage conditions were AC voltages of 5 V_pp_ and 10 V_pp_ at a frequency of 10 kHz, and the gradient of the electric field (∇|E|^2^) at a distance of 10 μm from the lower electrode was simulated using COMSOL Multiphysics (COMSOL Group). From the comparison of these two forces, it is anticipated that when the flow rate exceeds 1.0 μL/min at 10 V_pp_, the drag force will surpass the DEP force, leading to a decrease in recovery rate. Moreover, when the applied voltage is reduced to 5 V_pp_, even at lower flow rates such as 0.2 μL/min, more particles are expected to be lost due to drag force. Experimentally, it was confirmed that at flow rates exceeding 1.0 μL/min and voltages lower than 10 V_pp_, very few particles were trapped. To validate the potential of the proposed microfluidic device, trapping experiments of PS particles and *E. coli* were conducted at a flow rate of 1.0 μL/min and an applied voltage higher than 10 V_pp_, resulting in stable and high recovery rates, with measurements performed for various particle concentrations.

**Figure 9:**
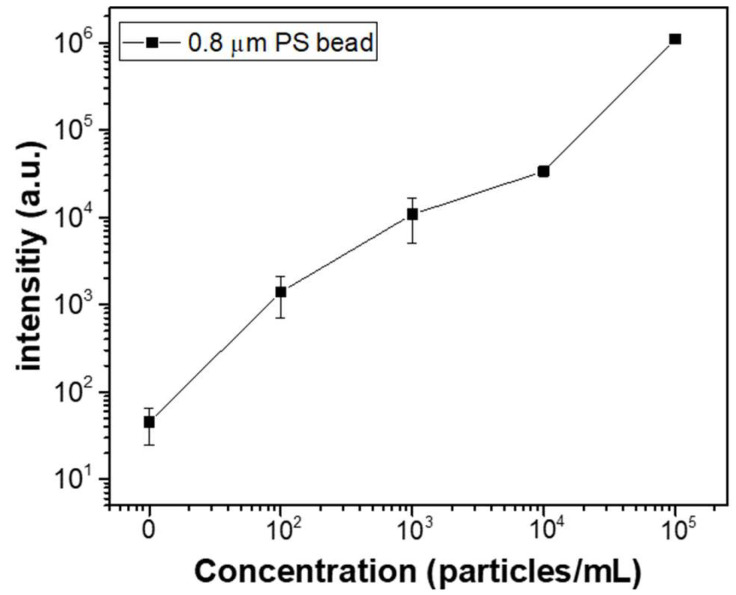
Fluorescence intensities corresponding to different microparticle concentrations.

**Figure 10:**
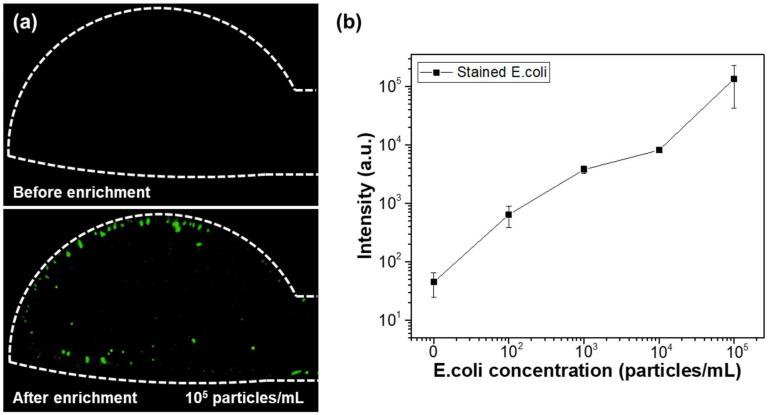
(**a**) Fluorescence images of *E. coli* before and after enrichment. (**b**) Fluorescence intensity measurement results according to *E. coli* concentration.

Figure 9 illustrates the changes in fluorescence intensity corresponding to different microparticle concentrations. The input AC signal was set to 20 V_pp_ at 10 kHz. Over a 50 min period, 0.8 μm fluorescent beads at concentrations of 10^2^, 10^3^, 10⁴, and 10⁵ particles/mL were injected at a flow rate of 1.0 μL/min, and their fluorescence intensities were measured and compared. To measure the fluorescence intensity for each particle concentration, three separate microfluidic devices were fabricated for each concentration, and the fluorescence intensity was measured for each device, with the results shown as averages and standard deviations. The measured average fluorescence intensities for DI water, 10^2^, 10^3^, 10⁴, and 10⁵ particles/mL were 45, 13.9 × 10^2^, 10.8 × 10^3^, 33.5 × 10^3^, and 10.9 × 10⁵, respectively. These results confirm that the proposed microfluidic chip allows for effective differentiation of fluorescent particle concentrations using fluorescence microscopy. Additionally, the same experiment was applied to bacteria. Fluorescently stained *E. coli* was used for fluorescence observation, with concentrations of 10^2^, 10^3^, 10^4^, and 10^5^ particles/mL. The solution was injected at a flow rate of 1.0 μL/min for 50 min, and the applied AC signal was 20 V_pp_ at 10 kHz. Figure 10a compares the fluorescence images of *E. coli* bacteria before and after 50 min of enrichment at a concentration of 10^5^ particles/mL. As shown in the figure, bacteria are concentrated in the electrode region on the membrane filter, where DEP forces are generated. Figure 10b presents the fluorescence intensity results after enrichment, based on the *E. coli* concentration. As with the particle concentration measurements, three separate microfluidic devices were fabricated for each bacterial concentration, and the results are shown. The results demonstrate a linear increase in fluorescence intensity with increasing bacterial concentration. Compared to the PS bead experiment, the fluorescence signal in the bacterial experiment was relatively lower. This difference is attributed to the variation in fluorescence intensity per particle between the fluorescent beads and stained bacteria.

In the bacterial concentration experiments, including the bacterial enrichment phase, the total detection time was approximately one hour. Throughout this period, no issues (such as variations in particle flow within the device or deformation of the membrane filter) were observed when flow rates between 1.0 and 2.0 μL/min were applied. Regarding the input voltage used to generate DEP forces, while increasing voltage can improve particle concentration efficiency, higher voltages can induce electrolysis of the fluid around the electrodes. This may lead to electrode damage or bubble formation, which in turn complicates optical measurements and significantly degrades device performance. In this study, when a voltage greater than 35 V_pp_ was applied, bubble formation due to electrolysis occurred, rendering fluorescence signal measurements impossible. The experimental results validate the potential of the microfluidic device proposed in this study for the concentration of particles, such as bacteria, and demonstrate its applicability in effective optical measurement and analysis.

The optical measurement method facilitated by the proposed device provides a detection time of approximately one hour and is capable of detecting significant fluorescence signals at low concentrations, as low as 10^2^ particles/mL. Previous studies that utilized membrane filters for bacterial detection [45,46] and DEP forces for detecting bacterial concentration [47,48,49,50] reported successful detection at concentrations in the range of 10^2^–10^3^ particles/mL. These studies applied efficient optical systems and various optical measurement techniques for bacterial detection. Expanding upon the research presented here, future studies could further enhance the microfluidic device’s performance by optimizing the optical system, incorporating fluorescent antibodies, or integrating alternative optical measurement techniques, such as SERS (Surface-Enhanced Raman Spectroscopy), to improve the precision and sensitivity of bacterial concentration measurements.

## 5. Conclusions

This study presents a novel microfluidic device that combines DEP forces with a membrane filter to efficiently concentrate and trap microparticles in a narrow region for enhanced optical measurement. Experimental results demonstrated the device’s effectiveness in trapping and concentrating microparticles, such as PS beads and bacteria, with fluorescence intensity increasing proportionally to particle concentration. Furthermore, when the DEP forces were applied to a small region of the membrane filter, the fluorescence signal increased significantly compared to experiments where DEP was not used, confirming that DEP significantly enhances particle concentration in the target region. Additionally, the device showed promising results for bacterial detection, particularly for *E. coli*, with a linear increase in fluorescence intensity with increasing bacterial concentration. The ability of the DEP-enhanced membrane filter to concentrate particles in a narrow region for sensitive optical analysis represents a significant improvement in particle trapping and detection. Future work will focus on optimizing the device for higher capture efficiency and exploring its potential for broader applications in fields such as environmental monitoring and biotechnology.

## Data Availability

The original contributions presented in this study are included in the article. Further inquiries can be directed to the corresponding authors.
